# Robotic endoscopy. A review of the literature ^1^

**DOI:** 10.1590/s0102-865020200020000006

**Published:** 2020-04-27

**Authors:** Thiago Arantes de Carvalho Visconti, José Pinhata Otoch, Everson Luiz de Almeida Artifon

**Affiliations:** I MD, Department of Gastroenterology-Endoscopy, School of Medicine, Universidade de São Paulo (USP), Brazil. Acquisition of data, manuscript preparation.; II Full Professor, Department of Surgery, School of Medicine, USP, Sao Paulo-SP, Brazil. Manuscript writing, critical revision, final approval.

**Keywords:** Endoscopy, Robotics, Endoscopic Mucosal Resection

## Abstract

**Purpose:**

To present new endoscopic robotic devices in the context of minimally invasive procedures with high precision and automation.

**Methods:**

Review of the literature by December 2018 on robotic endoscopy.

**Results:**

We present the studies and investments for robotic implementation and flexible endoscopy evolution. We divided them into forceps manipulation platforms, active endoscopy and endoscopic capsule. They try to improve forceps handling and stability and to promote active movement.

**Conclusion:**

The implementation and propagation of robotic models depend on doing what the endoscopist is unable to. The new devices are moving forward in this direction.

## Introduction

Endoscopy began as a gastrointestinal (GI) diagnosis method and became an important treatment method for GI pathologies nowadays. The equipment is in constant evolution, since the implementation of the electrical lamp, from the coming of flexible endoscopes, incorporation of ultrasonography and the recent development of robotic methods.

Endoscopic instruments had already been used for the urethra, bladder and uterine cervix. However, it was Adolf Kussmaul, in 1868, who performed the first direct esophagogastroscopy. After attending a sword-swallower performance, he demonstrated that it was possible to introduce a rigid tube to the stomach if head and neck hyperextended, yet without enough illumination. Joseph Leiter, in 1882, included an electrical lamp on the tip of the endoscope.

Endoscopy as known today is due to the invention of the flexible endoscope by Wolf and Schindler in 1932, spreading the diagnostic endoscopy use. The emergence of videoendoscopy expanded its use in the GI pathologies treatment^1 , 2^ .

With the evolution and propagation of endoscopy, two major dilemmas emerged. Both the endoscopic submucosal dissection (ESD), resecting lesions each time wider and more complex, and the willingness to perform Natural Orifice Transluminal Endoscopic Surgeries (NOTES) bring the need of platforms that promote stability and forceps manipulation that conventional equipment does not. Adding up to that, there is the pursuit of automation for equipment to do what, nowadays, only the endoscopist physician is capable of.

Thereby, the endoscopic robotic techniques can be divided into those developed to improve forceps handling and stability and those with active movement.

## Robotic flexible endoscopy

Flexible endoscopy is widely used for GI diagnosis and therapy, as it is little invasive and fast. Done by only one endoscopist and, most times, without general anesthesia^3^ . However, with the advance of its therapeutic purpose, the time and complexity of procedures have been drastically increasing, highlighting the operational limitations of flexible endoscopes. They have limitations regarding stability and forceps movement, with little possible angulation.

Robotics has more degrees of freedom to improve triangulation and traction precision for dissections and NOTES^3 , 4^ . Degrees of freedom are specific, defined modes in which a mechanical device or system can move. The number of degrees of freedom is equal to the total number of independent displacements or aspects of motion. Beyond that, it is necessary to keep searching for lower adverse events rates, pain and discomfort while the exam to promote higher patient acceptance.

The larger researches and investments in robotic endoscopy are directed to:

- Platforms capable of high degrees of freedom on forceps manipulation for ESD and NOTES^5^ .
- Active introduction of the endoscopes to reduce the influence of the operator ability and to reduce the discomfort and pain referred by the patients^8 , 9^ .
- Endoscopic capsule evolution to use it as screening for GI pathologies and as a therapeutic method^10 , 11^ .

## Forceps manipulation platforms

For ESD, it is necessary an adequate mucosal traction towards the lumen to expose the submucosal layer. The submucosal layer is dissected carefully, with hemostasis, until complete resection of the lesion. Mucosal traction needs to be constantly reallocated, due to angles and curves, especially in larger lesions. Those steps can take hours and bring technical difficulty. A range of instruments was developed to help the dissection, as different types of endoscopic knives and techniques to promote traction as cap-assisted^12^ ; metallic clips tied to strings^13 , 14^ and forceps attached to external channels^15 , 16^ . However, each one has its limitations. The cap-assisted technique reduces the vision field and sometimes does not promote the necessary traction. The clip is not possible to be pushed. Forceps in external channels can only perform traction in the same axis as the endoscope^17^ .

The robotic systems facilitate the techniques described above. The main principle of the majority is two attached arms in the tip of the endoscope, enabling the forceps manipulation to various directions with better triangulation, traction, exposure and dissection of the tissue^3^ . We will describe some of the devices that are being carried out and improved.

## Master and Slave Transluminal Endoscopic Robot (MASTER)

**Figure 1:**
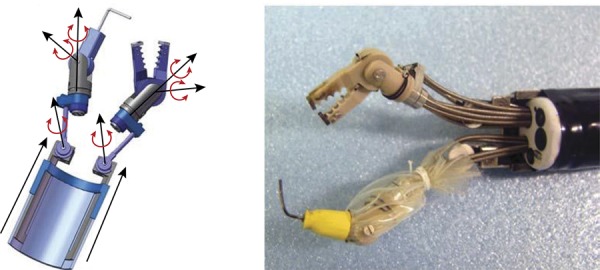
MASTER platform tip.18

**Figure 2:**
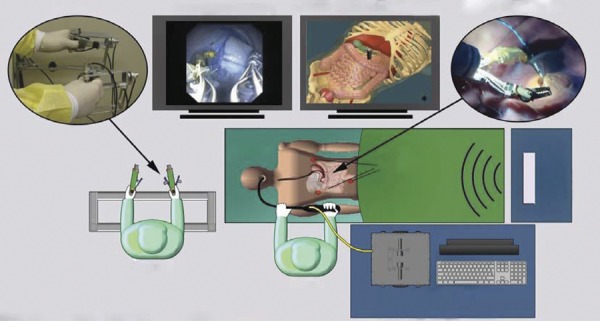
- Physicians disposal on the MASTER.18

Developed by the Nanyang Technological University and the National University of Singapore, the MASTER system consists of two attached arms to a conventional double-channel endoscope with a forceps and an electrocautery hook. It allows nine degrees of freedom ( Fig. 1 ). It is necessary two endoscopists to manipulate ( Fig. 2 ). The surgeon controls seven degrees of freedom and the endoscopist introduces the set until the desired GI local and controls the positioning and orientation, besides the other two degrees of freedom, that are not motorized^18^ . Animal studies demonstrated effectiveness on ESD, full-thickness gastric resection and hepatic resection^18^ . This system was used on human for a few gastric, one esophageal and one colon ESDs^23 , 24^ .

The narrow space interferes with the forceps manipulation, but en bloc resection was possible without major complications. Phee *et al* .^23^ evaluated five early gastric lesions restricted to the body or antrum. The mean time of margin free resection was 18 min (3-50 min), without adverse events. Although its viability, the MASTER has a few limitations. It is not possible to change the forceps, it is necessary to insert an overtube in the esophagus and the external control unit is big and restricts its displacement^25^ . The MASTER development continues to improve flexibility, precision and to promote kinetic sensation^26^ .

### STRAS/Anubiscope TM

**Figure 3:**
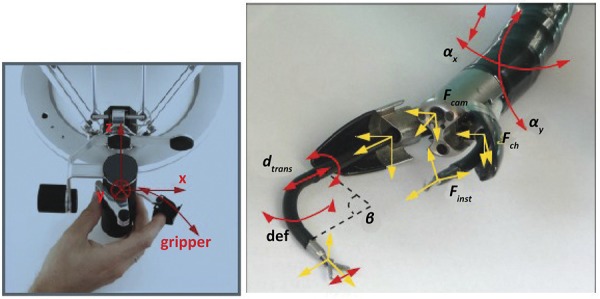
Manipulation, tip and degrees of freedom of AnubiscopeTM.27

**Figure 4:**
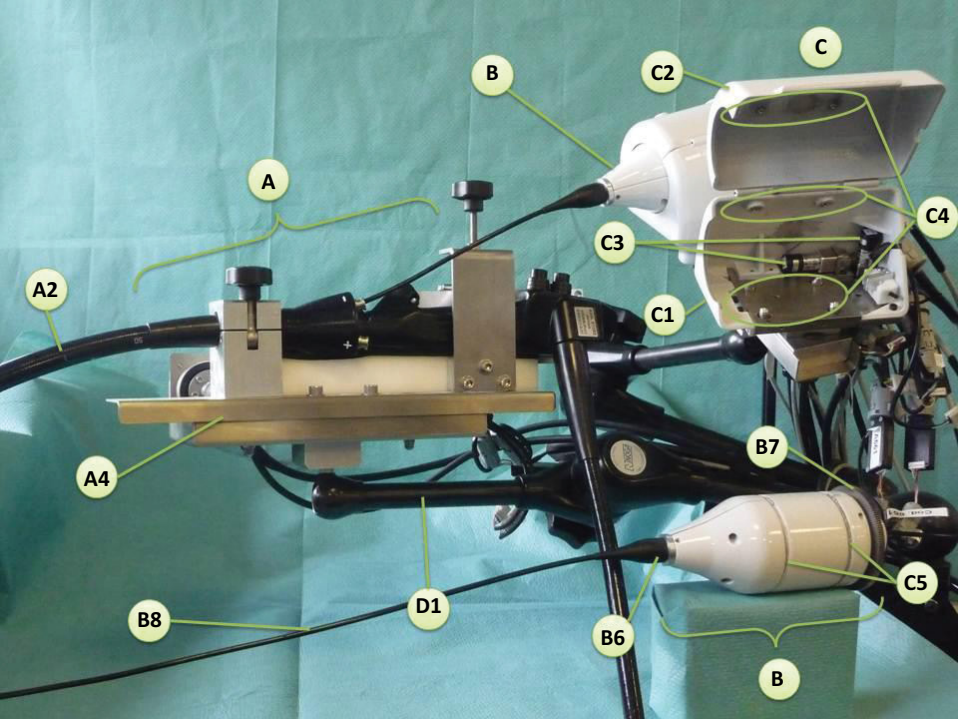
- STRAS operation module.27

STRAS is the robotic version of Anubiscope^TM^. Developed by the Research Institute against Digestive Cancer (IRCAD) with Karl-Storz, it is a modular system with a 16mm diameter endoscope. It has two 4,3mm diameter channels and one 3,2mm central channel. Specific forceps are capable of bounding at the tip, promoting rotation and translation with 10 degrees of freedom^7 , 25^ . A trocar like the tip of the endoscope protects the esophagus during the insertion and opens like a shell when it is in place. External traction wires electronically control the instruments, eliminating the resistance sensation of the initial Anubiscope, which had mechanical control^7 , 27^ . NOTES cholecystectomy was performed successfully by Anubiscope^TM^ in one patient^28^ . Anubiscope^TM^ needs good cooperation and synchrony between at least two physicians who share the workspace at the platform. In this sense, the robotics and telemanipulation provide the possibility of only one person controlling the entire equipment ( Fig. 3 ). The STRAS was developed to act as a teleoperated modular platform to eliminate the need for a second physician. The insertion of the scope is manual, but the surgical part of the procedure is teleoperated by STRAS. Zorn *et al* .^7^ reported twelve successful ESDs for large lesions in porcine models ( Fig. 4 ).

### EndoSAMURAI (Olympus Medical Systems Corp, Tokyo, Japan)

**Figure 5:**
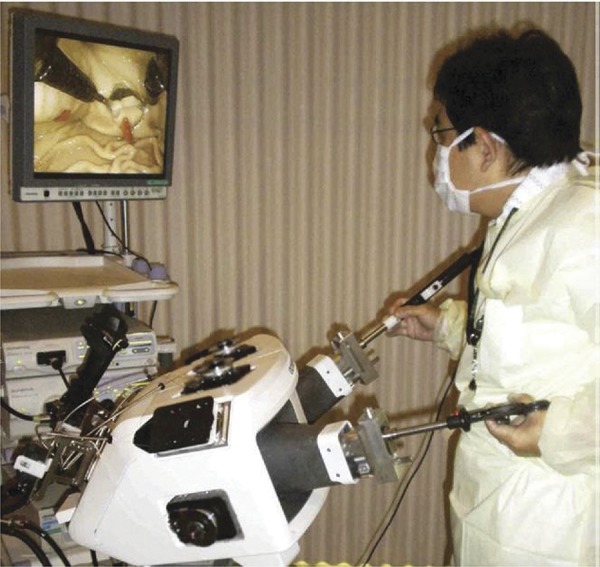
- EndoSAMURAI command console.29

**Figure 6:**
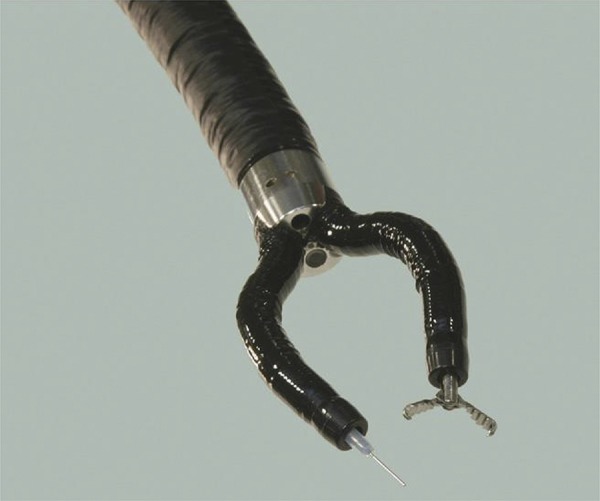
EndoSAMURAI tip.29

Developed by Olympus Medical Systems (Tokyo, Japan) for the use in NOTES. It is a system composed by a main body (command console) and the tube. The tip has two articulated arms with five degrees of freedom (up-down, right-left, forward-backward, open-close and rotation), besides a conventional work channel. The tube has 15mm diameter and the articulated arms have 2.8mm diameter. An exclusive overtube is required for the insertion of the tube. Two endoscopists are required for its manipulation, one at the tube and one at the command console managing the two articulated arms. The application in humans still needs validation, although its use has been demonstrated in ex vivo studies^6 , 29^ ( Figs. 5 and 6 ).

### Scorpion shaped endoscopic robot

**Figure 7:**
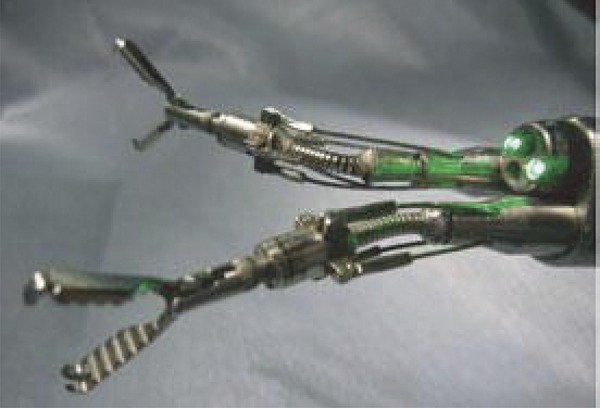
- Scorpion tip.30

Developed for NOTES and single port surgeries, it has two robotic arms controlled by external traction cables and a camera between the arms. One operator controls the tube and the other controls the robotic arms. One of its biggest advantages is the kinetic sensation on the arms manipulation. No studies reporting its viability in animals or humans have been published^30^ ( Fig. 7 ).

## Active endoscopy

Robotic assisted colonoscopy intends to improve the patient’s exam tolerance, to reduce pain, to reduce perforation risk and to promote cecal intubation regardless of the endoscopist ability. For those, it is necessary the colonoscope to have active motion and to mold to the colon. With robotic assistance, the physician could manipulate the colonoscope within a certain distance^2^ . The existing disposals have different insertion tactics. The majority uses inchworm-like movements or techniques derivate from balloon enteroscopy^3^ .

### Aer−O−ScopeTM

**Figure 8:**
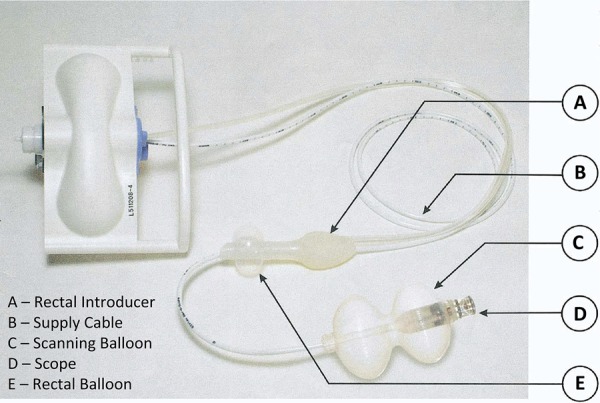
Aer−O−ScopeTM.31

**Figure 9:**
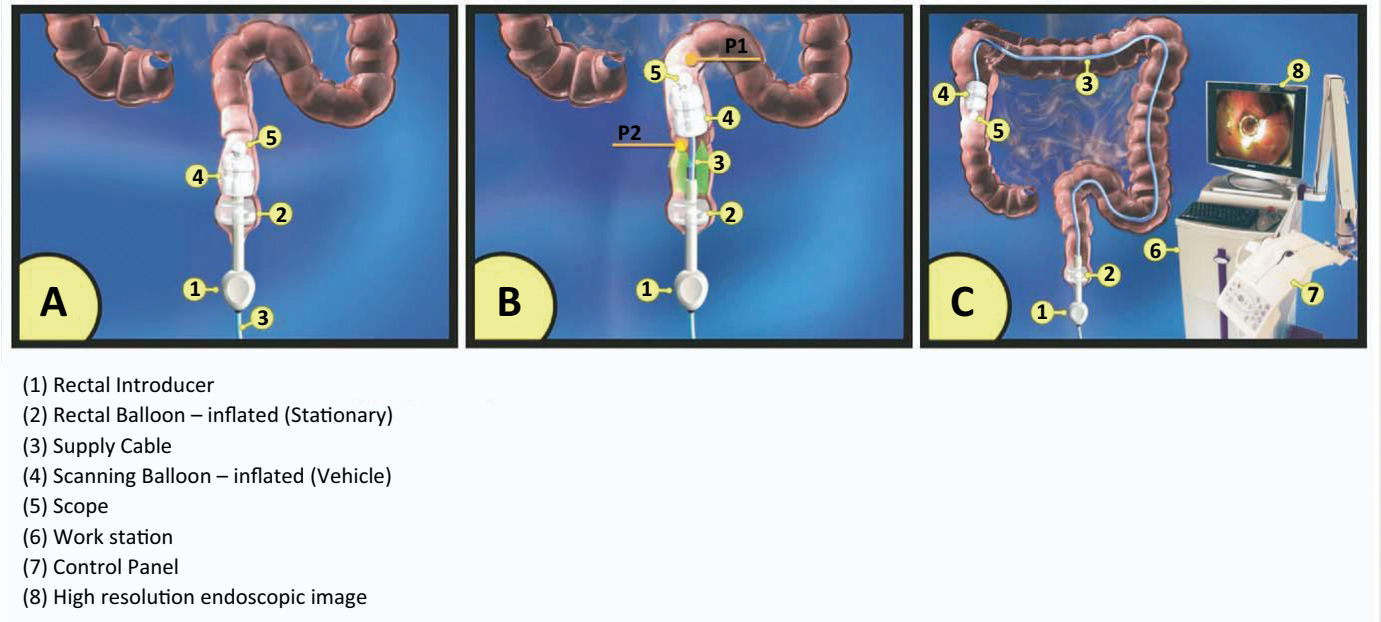
Aer−O−ScopeTM operation.31

A control station and disposable components compose the Aer−O−Scope^TM^. The disposable components are a rectal introducer, a supply cable and the optical capsule wrapped by a vehicle balloon. The rectal introducer is a silicone tube with a balloon attached to avoid air loss^31^ . After its introduction trough the anal canal, the remaining dispositive is inserted. The two balloons are insufflated and CO_2_ is insufflated between them. The pneumatic force applied in the bowel pushes the balloon forward, while the introductory balloon stays in the rectum. The pressures in the balloons and the bowel (before and after the balloon) are constantly measured and transmitted to the workstation. A computer algorithm adjusts the three pressures to advance the vehicle balloon and to avoid perforations. Once the system is in the cecum, the pressures are changed to maintain the colon distended for evaluation and the balloon regression to the rectum. The camera provides a circumferential view of 360º^32^ . Gluck *et al* .^33^ reported a cecal intubation rate of 98.2% ( Figs. 8 and 9 ).

### Endotics System (ERA Endoscopy S.r.l., Pisa, Italy)

Endotics system relies on the inchworm-like movement. A disposable probe has a manipulated movable tip and a flexible body controlled by a physician at the workstation.

**Figure 10:**
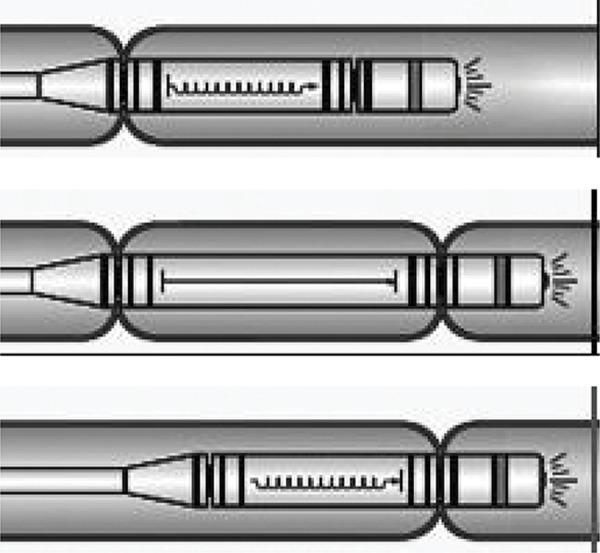
- Endotics movement mechanism.34

The proximal and distal dispositive can attach at the mucosa and an extension and retraction mechanism between them promote the insertion of the instrument like an inchworm^34^ . At first, the cecal intubation rate was only 27%. However, more recent studies reported a rate of 81.6%, still lower than the control group of 94.3%. Nevertheless, the pain and need for sedation are minimum^8 , 34^ ( Fig. 10 ).

### NeoGuide Endoscopy System (Neoguide Systems Inc., Los Gatos, CA)

**Figure 11:**
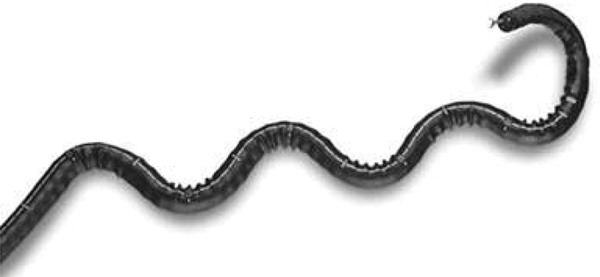
Independent movable segments controlled by NeoGuide sytem.35

The NeoGuide Endoscopy System is an articulated colonoscope controlled by a computer console developed to maintain the natural loops of the colon during insertion. Sensors at the tip and external detect the instrument position. The segments of the tube are independent and movable and they are electronically controlled. While the physician introduces the tube, it is shaped by the computer console according to the natural loops. Eickhoff *et al* .^35^ reported cecal intubation in 10 of 11 patients in a small human trial ( Fig. 11 ).

### Invendoscope: (Invendo Medical, Kissing, Germany)

**Figure 12:**
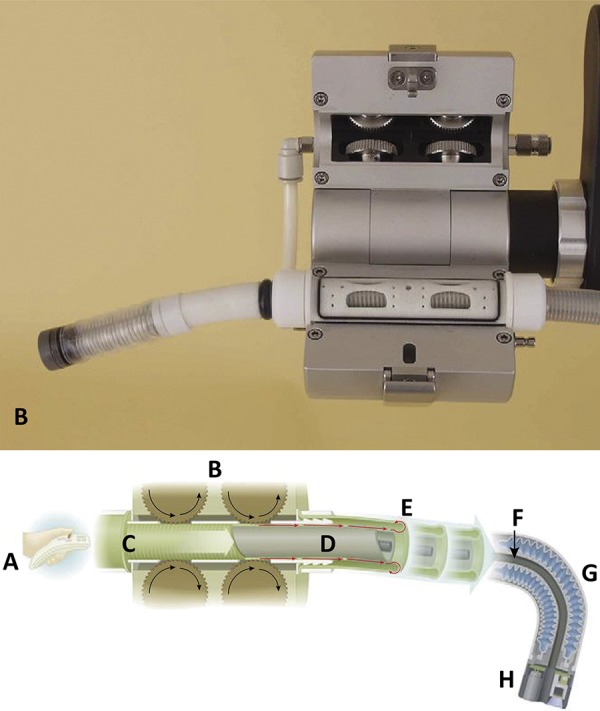
Invendoscope propulsion mechanism.36

Invendoscope is a single use, portable, engine driven colonoscope. Eight wheels out of the patient make the tube propulsion, controlled by a joystick and the physician. The tube diameter is 10mm and the length is 170 cm to 210 cm, depending on the version. A double inverted sleeve protects the tube and it is unrolled while it is inserted, serving as propulsion. Initial papers reported a cecal intubation rate of 82%. The failure causes were intense pain and impossibility to transpose the hepatic flexure^36^ . Groth *et al* .^9^ reported 61 patients with a cecal intubation rate of 98.4%, and a 15 min mean time. Only three patients needed sedation ( Fig. 12 ).

### EOR (Endoscopic Operation Robot)

The third version of the Endoscopic Operation Robot is a system attached to a conventional scope capable of manipulating it through a joystick with one hand. It is composed of a rotating handle, a load cell, a rotary motor, a torque sensor, and a joystick. It intends to replace one endoscopist when using multitask endoscopic systems as MASTER and EndoSamurai that requires at least two physicians. There are no studies in humans yet, only in models^37^ .

## Endoscopic capsule

The use of endoscopic capsules, established in the last two decades, represents an appealing alternative to traditional endoscopic techniques for gastrointestinal screening for its lack of discomfort and need for sedation. However, the current models are passive devices that depend on intestinal mobility, and it is not possible to control the camera direction.

Thereby, they are nowadays used mainly in the investigation of the small intestine in occult bleeds, since the small intestine has a virtual lumen that does not need insufflation for inspection, besides not possessing therapeutic abilities^38^ . Own locomotion system or external command needs to be developed to expand the application of capsules, and they are reported for the diagnosis of pathologies of the stomach and esophagus^39^ . When applied to the study of colon, robotic endoscopic capsules may overcome the pain and discomfort drawback of conventional colonoscopy, but still lack reliability, diagnostic accuracy and they fail to perform therapeutic functions at the same time^38 , 40^ .

A robotic endoscopic capsule platform should consist of six modules: locomotion, location, vision, telemetry, energy, and diagnostic and therapeutic tools. However, most capsules developed to date have only a few of these functions^38^ .

The active locomotion of the capsules can be accomplished by the capsule itself (through flapping tails, “legs”, “paddles” or propellers) or externally by magnetism^2 , 41^ . One of the greatest difficulties in achieving self-propulsion of capsules is the durability of their energy module since the batteries need to be too small to fit inside the capsule^10 , 38^ . Externally driven locomotion is more feasible and uses magnets for the creation of force fields that interact with magnetic components within the capsule; in this way, the presence of locomotion components in the capsule or batteries is not necessary.

## General considerations and perspectives

We present the researches and investments in the implementation of robotics and the evolution of flexible endoscopy. There is a search for systems that bring stability and greater controllability of the instruments for complex ESDs and NOTES. However, the new models presented have not yet been tested in large and challenging lesions, where they would show their full capacity and initial purpose, reducing technical difficulty and procedure time. For the implementation and diffusion of the robotic models, they must perform tasks that the endoscopist is incapable of, not only to reproduce what is already widely done. To move in this direction, the available models need to be constantly developed. Now to improve handling and stability; decrease the caliber and size of parts; and promote tactile sensation. However, this mission is not simple. Improved handling means increasing degrees of freedom, increasing the number of parts and instruments, making it harder to reduce the size of models.

Regarding active endoscopy, it is still necessary to develop models that prove safe and effective progression of the devices. The ability to perform small therapies, such as forceps polypectomies, also needs to be incorporated into these models. The endoscopic capsule is still far from being able to carry out self-propulsion and therapy. However, with external handling modules, the diagnostic exam of the stomach is already feasible and may be an alternative for screening tests. The idea of performing exams without the need of the endoscopist is still far from being materialized. In some cases, the need for the skilled professional has even increased, as in distance manipulation forceps models, where two endoscopists are necessary: one at the tube and another at the control console. The association of artificial intelligence with the processing and interpretation of computer images will certainly increase the autonomy of endoscopy, but the models currently available are still in research and this is not a reality for the near future.

## Footnotes

1 Research performed at Endoscopy Unit, Department of Gastroenterology, School of Medicine, Universidade de São Paulo (USP), Brazil.
